# Palliative care in the emergency department: An observational study of doctors in KwaZulu-Natal

**DOI:** 10.4102/safp.v66i1.5860

**Published:** 2024-04-19

**Authors:** Nagaleswari Sriranganathan, David Morris, Laura Campbell, Richard Hift

**Affiliations:** 1 Department of Emergency Medicine, Division of Emergency Medicine, School of Clinical Medicine, University of KwaZulu-Natal, Durban, South Africa; 2 Department of Medicine, Faculty of Research, University of KwaZulu-Natal, Durban, South Africa; 3 Department of Clinical Medicine, University of KwaZulu-Natal, Durban, South Africa

**Keywords:** emergency medicine, palliative care, knowledge, attitude, survey

## Abstract

**Background:**

The World Health Organization advocates the early, appropriate provision of palliative care (PC) to patients throughout the life course. Patient consultations to the emergency department (ED) have been recognised as opportunities to initiate or optimise their PC needs. This study aimed to assess the knowledge of and attitudes towards PC among doctors at emergency physician staffed EDs in KwaZulu-Natal, South Africa.

**Methods:**

A cross-sectional survey was conducted between November 2021 and February 2022 for doctors employed out at emergency physician staffed EDs in KwaZulu-Natal, South Africa, using the validated Palliative Care Attitude and Knowledge questionnaire. The variables assessed were the self-rated and basic knowledge and attitudes towards core domains of PC. Ordinal data were compared using the *t*-test or ANOVA as appropriate, using MedCalc® Statistical Software version 22.009.

**Results:**

Of the 39 participants, the scores for the knowledge questions showed that 15.3% participants had good knowledge, 53.8% had fair knowledge and 30.7% had poor knowledge. Participants had either favourable (58.8%) or an uncertain (41.0%) attitude towards PC. No correlation was seen between the knowledge and attitudes scores (Spearman’s rho = 0.13, 95% CI –0.19 to 0.43, *p* = 0.43).

**Conclusion:**

There appears to be a deficit in knowledge of PC among doctors in the ED and a need for in-service training in PC for emergency care physicians.

**Contribution:**

This study provides new knowledge around PC practices at EDs in KwaZulu-Natal, South Africa.

## Introduction

Palliative care (PC) is viewed as a form of care that improves the quality of life of patients with life-threatening illnesses and assists patients and their families in meeting the challenges associated with life-threatening illness. This includes the prevention and relief of suffering by means of early identification and treatment of pain and attention to all the physical, psychosocial and spiritual challenges associated life-threatening illnesses.^1^ The global burden of illness and the corresponding need for PC are immense.^2^ Recognising these important factors of patient care, the World Health Organization (WHO) advocates the early, appropriate provision of PC to patients who will benefit from it.^1^

The holistic practice of palliative medicine requires continual educational and professional support and training should be in accordance with global evidence-based perspectives, with consideration of the patient and population’s cultural beliefs.^1,3^ Initiatives focusing specifically on PC in South Africa began in the 1970s, with the launch of several projects intended to develop training programmes and policies to standardise PC practice in South Africa and to assist care organisations in developing comprehensive services. In 1987, the Hospice Palliative Care Association was established by members of hospices in South Africa, who identified the need for a national body to share best practice.^4,5^

The practice of emergency medicine (EM) involves the treatment of a divergent patient population by performing rapid patient assessments and delivering appropriate emergency interventions, which may be lifesaving.^6^ At first sight, therefore, the philosophy of EM is very different from that of PC, concentrating on immediate intervention with the intention of saving life, rather than the acceptance of the inevitability of death and long-term preparations for that. In practice, however, patients with terminal illness frequently seek assistance in the emergency department (ED) for acute exacerbations of or deterioration in their condition.^7^

In many cases, it is possible to address the immediate problem and refer the patient elsewhere for further care.^8^ Other patients who present to the ED at the end of the illness may require end-of-life care in the ED itself. Both situations require knowledgeable, insightful and sympathetic care on the part of EM practitioners: coordination with appropriate services for those discharged and sympathetic and holistic attention to the physical, psychological, social and spiritual issues surrounding impending death for those who remain in the ED.^9^

The quality of PC is determined by hospital-level and national-level system factors, including knowledge of PC principles, and effective communication between the care provider, patient, family and other members of the PC team.^10^ Given the important intersection of EM and PC, it is important that EM physicians are adequately trained for the purpose.^11^ The first step in identifying educational and training targets to improve EM training in PC is a needs assessment.^12,13^ Exploration of the knowledge and attitudes of providers may also yield useful culture-specific information and is essential in developing and refining training for healthcare workers and in supporting them in practice.^14^

Numerous studies have explored knowledge of PC and attitudes towards it among healthcare workers.^6,10,15,16,17,18,19,20,21^ Studies have suggested that specific healthcare education for healthcare providers is lacking in many African countries.^10^ This is important given that there is evidence that healthcare providers demonstrate better knowledge of and attitudes towards PC after receiving specific training.^20^

A number of studies have looked at the PC competence of EM physicians.^16,21,22^ It has been found that inadequate knowledge, inaccessibility of PC services and a lack of protocols and PC teams and specialists contributed to unfavourable attitudes of many ED clinicians^16^; correspondingly, physician training in the principles of PC is needed to achieve high-quality PC provision, especially in the areas of non-pain related physical symptoms and psychological symptoms.^16,22^

There is currently no information regarding attitudes towards PC and knowledge of its principles among EM practitioners in KwaZulu-Natal, South Africa. Locally generated data are helpful to precisely characterise the health requirements of a given population.^2^ This information would be valuable in identifying deficiencies in training, resources or the culture of care among this group of practitioners.^2^ We therefore undertook this study to assess PC knowledge and the attitude towards PC among doctors working in emergency physician staffed EDs in KwaZulu-Natal, South Africa, using the palliative care attitudes and knowledge questionnaire (PCAKQ).^15,16^

## Research methods and design

### Subjects

A cross-sectional survey was conducted among medical officers and EM registrars (trainee specialists) working at those public-sector EDs headed by a qualified EM physician in KwaZulu-Natal, South Africa, between November 2021 and February 2022. These departments offer ongoing continuous medical training in topics relevant to EM for their staff and are situated at the General Justice Gizenga Mpanza Hospital (KwaDukuza), Edendale Hospital and Greys Hospital (Pietermaritzburg), Ngwelezane Hospital (Empangeni), and Port Shepstone Hospital (Port Shepstone). Sessional medical officers working at the study sites were not included in the study. Forty-two medical officers and 12 registrars were eligible for participation.

### Survey instrument

We used the PCAKQ, developed in Kuwait in 2019.^15,16^ This instrument focuses on the provision of PC to any patient with life-threatening illnesses, through the disease trajectory, by non-PC physicians and is useful in assessing the current knowledge of and attitudes among doctors towards PC service delivery. It assesses two constructs: attitude and knowledge. All items are measured on a 5-point Likert scale and participants were asked to show level of agreement to a given statement. A favourable attitude is defined as a total score >41, uncertain attitude 25–41 and negative attitude scored <25. The knowledge assessment included dimensions of self-efficacy and basic knowledge of PC. *Self-knowledge* explored participants’ self-rated competence on PC in their clinical practice. A 5-point Likert scale was used and participants were rated as follows: 5, excellent response; 4, very good; 3, good; 2, weak; 1-none. *Basic knowledge* reflected the philosophy and principles of PC, management of pain and other symptoms. This was assessed using multiple-choice items that included a correct option, three distractors and a *don’t know* option. Correct answer scored 1 point. A total score >75% was defined as good knowledge, 50% – 75% as fair knowledge and less than 50% as poor knowledge. The tool was adjusted slightly to suit the needs of South African practitioners, asking for detail of local qualifications and using generic rather than trade names (Online Appendix 1).

The questionnaire described in the original article^15^ differed from the downloadable version, and we identified three errors in the published options in the knowledge section. They include Questions 2.2.2, 2.2.5 and 2.2.10 in the basic knowledge section, which we excluded from the analysis. Our conclusions are not affected in the sense that knowledge was categorised by percentages rather than absolute scores. We informed the authors of that paper of the error.

### Data collection and analysis

Surveys were paper-based and self-administered. Participants remained anonymous. All participants provided an academic honesty declaration form (Online Appendix 2), confirming that the contents of the survey were unknown to them before completing it (Online Appendix 2). Data were collated on a Microsoft Excel spreadsheet. Total scores for knowledge and attitude were created by summing the responses to the questions in the questionnaire. Where responses to questions were missing, responses were imputed based on the responses to other questions in the section.

We correlated knowledge and attitude scores using Spearman’s test. Categorical data were compared using chi-square or Fisher’s test as appropriate. Ordinal data were compared using the *t*-test or ANOVA as appropriate, using MedCalc® Statistical Software version 22.009 (MedCalc Software Ltd, Ostend, Belgium; https://www.medcalc.org; 2023). We estimated that a sample size of 54 would allow us to estimate the proportion of respondents with good knowledge of PC to within 20%, using 95% confidence intervals and a baseline estimate of 50%.

### Ethical considerations

Ethical clearance to conduct this study was obtained from the University of KwaZulu-Natal, Biomedical Research Ethics Committee (No. BREC/00002522/2021). Gatekeeper permission was provided by the KwaZulu-Natal Department of Health. No personally identifiable data were requested by the analysed surveys, and all results were anonymous. All participants provided written informed consent.

## Results

### Participants

The total number of potential participants identified across all sites was 49, of whom 39 agreed to participate. This included 9 registrars and 30 medical officers. Seventeen were male (44%) and 22 were female (56%). The median clinical experience (including ED experience) of the participants was 5 years (IQR 3.3 to 7.6, range 0–21 years). Other characteristics are summarised in Table 1. No registrars are stationed at Greys Tertiary Hospital and Port Shepstone Regional Hospital. Registrars were significantly more likely than medical officers to possess a diploma in primary emergency care (DipPEC) (*p* = 0.000).

**TABLE 1:** A summary of participants.

Hospital	Medical officer		Registrar		Total	
	*n*	DipPEC (*n*)	*n*	DipPEC (*n*)	*n*	DipPEC (*n*)
GH	1	0	0	0	1	0
GJGMRH	5	0	2	1	7	1
HGRH	11	3	4	3	15	6
NGW	8	0	3	3	11	3
PSRH	5	0	0	0	5	0

**Totals**	**30**	**3**	**9**	**7**	**39**	**10**

Note: DipPEC, Diploma in Primary Emergency Care. The number in parentheses reflects the number of staff within that category who possess this diploma.

### Training in palliative care

Fifteen medical officers (50%) and 4 registrars (44%), comprising 49% of the total sample, reported having received some training in PC. The intensity of training received was extremely variable: from formal training for 4 weeks (1, [3%]), through 2 weeks (4, [10%]) and 1 week (1, 3 [10%]), to some exposure during continuous medical education (CME) activities (10 [26%]).

### Palliative care discussions with patients or families

Twenty-six subjects (67%) had participated in some discussion of PC with either patients or families in the preceding 3 months. Of these, 3 (8%) reported having participated in more than 15 such discussions and 2 (6%) in more than six.

### Results of the questionnaire

**FIGURE 1:**
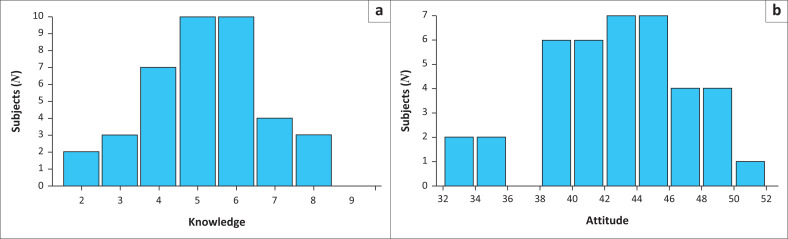
A summary of results obtained on the questionnaire in terms of (a) *Knowledge of palliative care* and (b) *Attitude towards palliative care.* The maximum possible score is on 9 and 55.

Results are summarised in Figure 1.

The mean score for the knowledge questions was 5.1 out of 9 (s.d. 1.5, range 2–8). Using the categories employed in the study by,^15^ 15.3% participants had good knowledge (score > 75%), 53.8% had fair knowledge (score 50% – 75%) and 30.7% had poor knowledge (score < 50%). The mean score for the attitude section was 42 (s.d. 4.2, range 32–50) out of a total possible of 55. Participants had either favourable (58.8%) or an uncertain (41.0%) attitudes towards PC. No participant had an unfavourable attitude towards PC. No correlation was seen between the knowledge and attitudes scores (Spearman’s rho = 0.13, 95% CI –0.19 to 0.43, *p* = 0.43).

Results are characterised by respondents in Table 2. Scores varied considerably across the hospitals: For knowledge, the four best-performing hospitals recorded means between 5.0 and 5.9, while the worst-performing scored 3.4 (*p* = 0.06). No difference was noticed between hospitals for attitude (*p* = 0.52).

**TABLE 2:** Breakdown of results by category of respondent.

Comparison groups	Knowledge					Attitude				
	Group A		Group B		*p*-value	Group A		Group B		*p*-value
	*n*	%	*n*	%		*n*	%	*n*	%	
MO (A) versus Registrar (B)	5.2	1.5	5.3	1.7	0.80	42.0	4.4	42.7	4.1	0.91
DipPEC (A) versus no DipPEC (B)	5.1	1.4	5.2	1.6	0.80	42.6	3.6	42.0	4.5	0.45
Received training (A) versus no training (B)	5.6	1.4	4.9	1.6	0.13	42.9	4.2	41.5	4.2	0.31

MO, Medical officer; DipPEC, Diploma in Primary Emergency Care.

## Discussion

The ED is a hub to which patients with various conditions present, often as a first point of contact with the healthcare system.^23^ Patients with life-threatening illnesses present to the ED regularly, for problems such as disease progression, infection, treatment-related complications and non-cancer-related problems. Other studies have found that while ED staff acknowledge the need to provide symptomatic relief to PC patients, there is uncertainty with regard to the extent of service delivery and expectations regarding the level of expertise required by the doctor to provide PC in the ED.^6^ A different skill set is required to manage patients with terminal conditions in the ED: traditional practices of resuscitation and stabilisation have been reported as inappropriate in most situations involving of end-of-life care.^24^ This is often a source of anxiety for doctors in the ED, who need to transition from a context of life-saving intervention to palliation.^6^ The lack of awareness about the spectrum of PC services, the lack of practice guidelines regarding symptom management and continuity of care and the absence of complete medical records of patients with preexisting illness may affect the initiation of PC in the ED and continuity of care after the ED visit.^10^

Inadequate clinician education and training have been shown to contribute to suboptimal PC delivery to patients and their families.^6^ We found that 30.7% of our participants had poor knowledge regarding PC concepts. This is not unexpected given experience elsewhere: 49% of ED physicians studied in the original PCAKQ study had poor knowledge,^15,16^ of PC among primary care physicians in the study by Hamdan, Yaacob,^18^ despite a generally positive attitude towards the subject. There are multiple possible reasons for this poor knowledge. The participant’s length of time since graduating from medical school has shown a significant positive effect on knowledge in some studies.^17,18^ This may be because of the introduction of compulsory undergraduate coursework in palliative medicine. Poor knowledge scores were associated with inadequate PC training and limited available guidelines for practice.^17,18,19^ Interestingly, we found no consistent improvement in knowledge as level of training in emergency care progressed. Scores did not appear to differ between subjects who had received some training versus those who had not, those with a basic medical degree and those with a DipPEC or between registrars and medical officers. The most likely explanation is that neither the syllabus for the DipPEC nor the Fellowship of the College of Emergency Medicine of South Africa (FCEM) places sufficient emphasis on PC, and the *ad hoc* training that some of our participants reported having received has not succeeded in imparting the necessary knowledge either.

Analysis of the individual questions within the PCAQK suggested that our participants had somewhat better knowledge of the treatment of pain than of other areas important to PC (data not shown). This may be because ED doctors are experienced in the use of potent analgesics, such as the opioids.^24^ As reported in other studies,^17,18^ however, we noticed weaknesses in knowledge of the management of other physical symptoms and of psychological issues.

Our participants had more favourable attitude (58.8%) towards PC than those in the original PCAKQ study (18.3%).^16^ The majority of participants agreed that PC services in the ED is an important aspect of patient care. Dissatisfaction with PC services, accessibility, length of coverage and communication between ED doctors, doctors in specialist departments and allied health services are systems-level issues identified in other studies that contribute to unfavourable attitudes.^16,25,26^ The ED environment provides limited privacy and time for discussions of PC, factors that have been identified as a barrier to respectful communication regarding end-of-life decisions with patients and their relatives.^19^ While previous studies have found an association between knowledge and attitude,^16,18,27^ we found that there was no correlation between the knowledge and attitudes scores (Spearman’s rho = 0.13, 95% CI –0.19 to 0.43, *p* = 0.43). Physicians with better knowledge had more positive attitudes in other studies, and it is suggested that training in PC concepts and symptom management contributed to better attitudes and willingness to provide PC when needed.^16,18,19,22,27^ Other factors that may contribute to positive attitudes are participants’ exposure in managing patients with terminal illness in their clinical practice and clinicians’ position in the care team.^28^ The need for PC services in the ED is growing and doctors in the ED are faced with patients in need of PC regularly. As such, doctors need to be capable of providing PC as part of their skill set.

There are several limitations in this study that could be addressed in future research. Firstly, the generalisability of the results is limited by the small sample size. Secondly, only staff working at public sector facilities were invited to participate. We believe that our results are broadly representative of the ED staff in these facilities, but the access to detailed patient records, access to allied healthcare facilities, availability of PC specialist teams and access to hospice facilities may contribute to better PC attitudes and practice in private sector. However, provision of PC depends on physician knowledge, training and referral pathways. Thirdly, the PCAKQ was developed in Kuwait and has not been previously applied or validated in South Africa. The PCAKQ explored a limited number of primary PC concepts around pain and non-pain related symptoms management. The downloadable version of the original PCAKQ had grammatical errors in the knowledge section of questionnaire, and this was identified during the analysis of the data. As a result, these questions were excluded in the data analysis, and some concepts were not represented in the data. This affected the interpretation of the data around PC knowledge possessed by participants. The questionnaire needs to be edited and validated should it be used again.

## Conclusion

To the best of our knowledge, this is the first study that explores the knowledge and attitude towards PC among South African emergency care physicians. We are encouraged to have shown that participants viewed PC as an important aspect of patient care, but we are concerned that knowledge of PC principles appears to be inconsistent, patchy and incomplete. There appears to be a deficit in knowledge of PC among both registrars and medical officers, and no evidence that successful completion of the DipPEC examination alleviates this. This raises concerns around the adequacy of current postgraduate training curriculum in emergency care. We suggest that the extent to which PC is included in the curricula for both the FCEM and the DipPEC is critically examined and its place improved in terms of both emphasis and structure if necessary. We also believe that there is a much greater need for in-service training in PC for emergency care physicians. Lastly, formal practice guidelines for PC provision in the ED would benefit ED doctors and result in improvement in the standard-of-care given to end-of-life patients and patients with unmet or unrecognised PC needs.
